# Disruption of Two-component System LytSR Affects Forespore Engulfment in *Bacillus thuringiensis*

**DOI:** 10.3389/fcimb.2017.00468

**Published:** 2017-11-03

**Authors:** Qi Peng, Jianbo Wu, Xiaomin Chen, Lili Qiu, Jie Zhang, Hongtao Tian, Fuping Song

**Affiliations:** ^1^State Key Laboratory for Biology of Plant Diseases and Insect Pests, Institute of Plant Protection, Chinese Academy of Agricultural Sciences, Beijing, China; ^2^Institute of Food Science and Technology, Hebei Agricultural University, Baoding, China

**Keywords:** two-component system, LytSR, sporulation, *spoIIP*, *Bacillus thuringiensis*

## Abstract

Two-component regulatory systems (TCSs) play pivotal roles in bacteria sensing many different stimuli from environment. Here, we investigated the role of the LytSR TCS in spore formation in *Bacillus thuringiensis* (*Bt*) subsp. *kurstaki* HD73. *lacZ* gene fusions revealed that the transcription of the downstream genes, *lrgAB*, encoding two putative membrane-associated proteins, is regulated by LytSR. The sporulation efficiency of a *lytSR* mutant was significantly lower than that of wild-type HD73. A confocal microscopic analysis demonstrated that LytSR modulates the process of forespore engulfment. Moreover, the transcription of the *lytSR* operon is regulated by the mother-cell transcription factor SigE, whereas the transcription of the sporulation gene *spoIIP* was reduced in the *lytSR* mutant, as demonstrated with a β-galactosidase activity assay. These results suggest that LytSR modulates forespore engulfment by affecting the transcription of the *spoIIP* gene in *Bt*.

## Introduction

As a type of specialized differentiated cell, spores are used by *Bacillus* to survive starvation and harsh conditions. *Bacillus subtilis* is the best-studied spore-forming bacterium. Its endospore is formed by an unusual mechanism involving asymmetric cell division, followed by the engulfment of the cells and the spore morphogenesis (Errington, 2003). The formation of the asymmetric septum is a key event in spore development. Two sigma factors, σ^F^ and σ^E^, are instrumental in setting the cell-specific programs of gene expression in motion. Some σ^E^- and σ^F^-dependent genes are also involved in the prespore engulfment process (Errington, 2003). σ^E^ is initially produced as an inactive pro-σ^E^ precursor and is specifically activated only in the mother cell. The σ^E^ regulon includes genes necessary for engulfment (Tan and Ramamurthi, 2014). During engulfment, peptidoglycan degradation machinery composed of SpoIID, SpoIIM, and SpoIIP is initially required for septal-wall thinning and subsequently for the movement of the engulfing membranes (Ohara et al., 2015). The completion of engulfment is a key event governing the later stages of spore development. In the prespore, a third sporulation-specific sigma factor, σ^G^, becomes active at this time, and this sigma factor controls the final stages of development inside the spore. The final mother–cell-specific sigma factor, σ^K^, is regulated at multiple levels and is involved in the formation of the spore coat and in spore maturation (Errington, 2003; Hilbert and Piggot, 2004).

The two-component regulatory system (TCS), which typically consists of a membrane-spanning histidine kinase (HK) sensor and a cytoplasmic response regulator (RR), also plays a critical role in bacterial adaptation, survival, and virulence by sensing changes in the external environment and modulating gene expression in response to a variety of stimuli (Skerker et al., 2005). Studies have found that the transition of *B. subtilis* from vegetative growth to sporulation is governed by the master transcription factor Spo0A, which is regulated by a complex phosphorelay involving five autophosphorylating histidine kinases (KinA–E), which respond to different types of environmental stress. Spo0A is not a simple TCS containing a kinase and a regulator. Phosphorylated Spo0A is an essential positive regulator of the initiation of sporulation (Burbulys et al., 1991; Jiang et al., 2000; Fujita and Losick, 2003). However, it is not known whether other TCSs are involved in the subsequent spore formation stage, which consists of asymmetric cell division and engulfment in *Bacillus*.

The *Bacillus cereus* group of closely related Gram-positive, spore-forming bacteria includes *B. cereus*, a common cause of human food poisoning, *B. thuringiensis* (*Bt*), an insect pathogen, and *B. anthracis*, the etiological agent of anthrax in mammals (Schnepf et al., 1998; Stenfors Arnesen et al., 2008). The general functions of some TCSs in *B. cereus* strains have been studied. For example, PP2C-type phosphatase RsbY receives its input from the multi sensor hybrid kinase RsbK, and RsbKY has been shown to regulate the activity of the alternative sigma factor B (van Schaik et al., 2005; de Been et al., 2010). SpsRK is active in response to glucose-6 phosphate and regulates the activity of the *spsABC* operon, which is involved in sugar phosphate transport (Song et al., 2012). In *B. anthracis*, LytSR regulates murein hydrolase activity, whereas the *lrgAB* genes, which are regulated by LytSR, affect stationary-phase survival and sporulation efficiency (Chandramohan et al., 2009). The parental strain has a sporulation efficiency of 88%, whereas the sporulation efficiency of the *lrgAB* mutant is only 5%, suggesting that the *lrgAB* gene products have a dramatic impact on sporulation in *B. anthracis* (Chandramohan et al., 2009). However, how LytSR affects sporulation remains unclear.

The functions of the LytSR TCS in *Bt* were investigated in this study using *Bt* subsp. *kurstaki* HD73. Our results show that the downstream genes, *lrgAB*, are regulated by LytSR, which is under the control of the mother cell transcription sigma factor SigE. LytSR modulates the subsequent forespore engulfment process and regulates the expression of the sporulation gene *spoIIP*.

## Materials and methods

### Bacterial strains, media, and DNA manipulation

The bacterial strains and plasmids used in this study are listed in Table 1. *Bt* strain HD73 (accession no. CP004069) was used in this study (Liu et al., 2013). The *Bt* strains were transformed by electroporation, as previously described (Lereclus et al., 1989). *Escherichia coli* and the *Bt* strains were cultured in Luria-Bertani (LB) medium or Schaeffer's sporulation medium (SSM, 8 g of nutrition broth, 0.12% MgSO_4_ [m/v], 0.1% KCl [m/v], 0.01 M NaOH, 0.1 M MnCl_2_, 0.01 M Ca(NO_3_)_2_, and 0.01 M FeSO_4_ in 1 L of H_2_O; Schaeffer et al., 1965) with shaking (220 rpm) at 37 and 30°C, respectively. The antibiotic concentrations used for bacterial selection were 100 μg/ml kanamycin and 10 μg/ml erythromycin for *Bt* and 100 μg/ml ampicillin for *E. coli*. DNA manipulation as previously described (Peng et al., 2014). Oligonucleotide primers were listed in Table 2.

**Table 1:** Strains and plasmids used in this study.

**Strains/plasmids**	**Relevant genotype and characteristics**	**Resource**
**STRAINS**		
*E. coli* TG1	Δ(*lac-proAB*) *supE thi hsd-*5 (*F' traD36 proA*^+^ *proB*^+^ *lacI*^q^ *lacZ*ΔM15), general purpose cloning host	Laboratory collection
*E. coli* ET 12567	*F*^−^*dam-13*::Tn*9 dcm-6 hsdM hsdR recF143 zjj-202*::Tn*10 galK2 galT22 ara14 pacY1 xyl-5 leuB6 thi-1*, for generation of unmethylated DNA	Laboratory collection
HD73	*B. thuringiensis* strain carrying the *cry1Ac* gene	Laboratory collection
HDΔ*sigE*	HD73 mutant type, Δ*sigE*	Du et al., 2012
HDΔ*lytSR*	HD73 mutant type, Δ*lytSR*	This study
HDΔ*lrgAB*	HD73 mutant type, Δ*lrgAB*	This study
HDΔ*spoIID*	HD73 mutant type, Δ*spoIID*	This study
HDΔ*spoIIM*	HD73 mutant type, Δ*spoIIM*	This study
HDΔ*spoIIP*	HD73 mutant type, Δ*spoIIP*	This study
Δ*sigE*(P*lytSR*)	HDΔ*sigE* carrying pHT304P*lytSR*	This study
HD(P*lytSR*)	HD73 carrying pHT304P*lytSR*	This study
Δ*lytSR*(P*lrgAB*)	HDΔ*lytSR* carrying pHT304P*lrgAB*	This study
HD(P*lrgAB*)	HD73 carrying pHT304P*lrgAB*	This study
Δ*lytSR*(P*spoIID*)	HDΔ*lytSR* carrying pHT304P*spoIID*	This study
Δ*lytSR*(P*spoIIM*)	HDΔ*lytSR* carrying pHT304P*spoIIM*	This study
Δ*lytSR*(P*spoIIP*)	HDΔ*lytSR* carrying pHT304P*spoIIP*	This study
Δ*sigE*(P*spoIID*)	HDΔ*sigE* carrying pHT304P*spoIID*	This study
Δ*sigE* (P*spoIIM*)	HDΔ*sigE* carrying pHT304P*spoIIM*	This study
Δ*sigE* (P*spoIIP*)	HDΔ*sigE* carrying pHT304P*spoIIP*	This study
HD(P*spoIID*)	HD73 carrying pHT304P*spoIID*	This study
HD(P*spoIIM*)	HD73 carrying pHT304P*spoIIM*	This study
HD(P*spoIIP*)	HD73 carrying pHT304P*spoIIP*	This study
Δ*lrgAB*(*lrgAB*)	HDΔ*lrgAB* genetic complementation strain carrying pHT*lrgAB* plasmid; Erm^r^	This study
Δ*lytSR*(*lytSR*)	HDΔ*lytSR* genetic complementation strain carrying pHT*lytSR* plasmid; Erm^r^	This study
**PLASMIDS**		
pMAD	Amp^r^, Ery^r^, temperature-sensitive *Bt*-*E. coli* shuttle vector	Arnaud et al., 2004
pHT304-18Z	Promoterless *lacZ* Vector, Erm^r^, Amp^r^	Agaisse and Lereclus, 1994
pHT315	*B. thuringiensis*-*E. coli* shuttle vector	Arantes and Lereclus, 1991
pHT*lytSR*	pHT315 with *lytSR* genetic complementation fragment	This study
pHT*lrgAB*	pHT315 with *lytAB* genetic complementation fragment	This study
pMAD-Δ*lytSR*	pMAD with *lytSR* deletion fragment	This study
pMAD-Δ*IrgAB*	pMAD with *lrgAB* deletion fragment	This study
pMAD-Δ*spoIID*	pMAD with *spoIID* deletion fragment	This study
pMAD-Δ*spoIIM*	pMAD with *spoIIM* deletion fragment	This study
pMAD-Δ*spoIIP*	pMAD with *spoIIP* deletion fragment	This study
pHT304P*spoIID*	Amp^r^, Erm^r^, pHT304–18Z carrying promoter upstream from *spoIID*	This study
pHT304P*spoIIM*	Amp^r^, Erm^r^, pHT304–18Z carrying promoter upstream from *spoIIM*	This study
pHT304P*spoIIP*	Amp^r^, Erm^r^, pHT304–18Z carrying promoter upstream from *spoIIP*	This study
pHT304P*lytSR*	Amp^r^, Erm^r^, pHT304–18Z carrying promoter upstream from *lytSR*	This study
pHT304P*lrgAB*	Amp^r^, Erm^r^, pHT304-18Z carrying promoter upstream from *lrgAB*	This study

**Table 2:** Sequences of oligonucleotide primers used in this study.

**Primer name**	**Sequence (5′ → 3′)^a^**
*lytSR*-1F	CGC**GGATCC**AACTCCCATTCCAACTAA
*lytSR*-1R	CTCAAATGGTTCGCTGGTAGTTGGAGTTGTAAC
*lytSR*-2F	GGAAATACGATTATGTGACGATGAAATGTTAGCACGTGAT
*lytSR*-2R	CG**GAATTC**GTGATTCAACTTGCTCCA
*lrgAB*-1F	CG**GGATCC**GGCATGAAATGATCTAATTTGCGGG
*lrgAB*-1R	CTCAAATGGTTCGCTGGTAGTTGGAGTTGTAAC
Kan-F	GTTACAACTCCAACTACCAGCGAACCATTTGAG
Kan-R	CATATTCTCAGCTATTATGAAATTCCTCGTAGGCGC
*lrgAB*-2F	GCGCCTACGAGGAATTTCATAATAGCTGAGAATATG
*lrgAB*-2R	CG**GAATTC**GAAACGAAGCACGAAATAAAGGGGAC
*lrgAB*hf-F	AA**CTGCAG**CGCAAATAGAAACGAAGCAC
*lrgAB*hf-R	CG**GGATCC**C TTACTATCCAATGAATGGTATG
*lytSR*hf-F	ACGC**GTCGAC**CAGTAAGATTGTGAAGGCCATTG
*lytSR*hf-R	CG**GAATTC**TTAAATACGAAGCAGCTTCTTGAG
*spoIID*-1F	GGCGATATC**GGATCC**CCGGATTATGAATCATCATTCGTCC
*spoIID*-1R	CTCAAATGGTTCGCTGACGATGAATGATTATG
kanD-R	CTCTTAATAGCGCTCAAATTCCTCGTAGGCG
kanD-F	CATAATCATTCATCGTCAG CGAACCATTT GAG
*spoIID*-2F	CGCCTACGAGGAATTTGAGCGCTATTAAGAG
*spoIID*-2R	CGGGAGCTC**GAATTC**GAACGGTCCAAACAGCTTACAAGGTG
*spoIIM*-1F	GGCGATATC**GGATCC**CACCTTAAAGCTCCAGTCTCGTTCTACTTTC
*spoIIM*-1R	CTCAAATGGTTCGCTGAAAG AAGTCGTTGAGG
kanM-F	CCTCAACGACTTCTTTCAG CGAACCATTT GAG
kanM-R	CATTTTATTTACAACGTAAATTCCTCGTAGGCGC
*spoIIM*-2F	GCGCCTACGAGGAATTTACGTTGTAAATAAAATG
*spoIIM*-2R	CGGGAGCTC**GAATTC**GAACGGTCCA AACAGCTTACAAGGTG
*spoIIP*-1F	GGCGATATC**GGATCC**GCGGAAGTACCATGTGGCTGTAATAAGG
*spoIIP*-1R	CTCAAATGGTTCGCTGAAAG AAGTCGTTGAGG
kanP-R	CAAATGCTTTAGCAAGAAATTCCTCGTAGGCG
kanP-F	GTTATTACTACAATGCTACAG CGAACCATTTGAGG
*spoIIP*-2R	CGGGAGCTC**GAATTC**CCAATACCTCGCCCGTTATACTCTTGC
*spoIIP*-2F	CGCCTACGAGGAATTTCTTGCTAAAGCATTTG
*spoIID*-F	CCTGTCACATACTCCTCCAC
*spoIID*-R	AGCCCTTGTTATTCCATTT
*spoIIP*-F	CAACTAGAAGGAGAAGGGAT
*spoIIP*-R	TTCTTTCGGGCACTATCA
*spoIIM*-F	ATGCCTAATCATCCGTAA
*spoIIM*-R	AAAAGGAGTTGTCGTTGG

a *Restriction sites are underlined and in bold font*.

### Construction of *lytSR* and *lrgAB* mutants

DNA fragments corresponding to the downstream and upstream regions of the *lytSR* genes (HD73_5856 and HD73_5855) were amplified by PCR using chromosomal DNA from *Bt* HD73 as the template and the *lytSR*-1F/*lytSR*-1R and *lytSR*-2F/*lytSR*-2R primer pairs, respectively. The corresponding DNA fragments were fused with overlapping PCR using primers *lytSR*-1F and *lytSR*-2R, and the PCR product was digested with *Bam*HI and *Eco*RI. The fragments were purified and ligated with the temperature-sensitive suicide plasmid pMAD (Arnaud et al., 2004) digested with the same enzymes, to yield the recombinant plasmid pMAD-Δ*lytSR*, which was used to transformed into host strains with electroporation. The confirmed transformants were incubated at 39–41°C. Colonies lacking erythromycin resistance were selected and one mutant strain, HDΔ*lytSR*, was verified with PCR.

The upstream (562-bp) and downstream (561-bp) fragments of *lrgAB* (HD73_5854 and HD73_5853) were PCR amplified with the primer pairs *lrgAB*-1F/*lrgAB*-1R and *lrgAB*-2F/*lrgAB*-2R, respectively, and using *Bt* HD73 genomic DNA as the template. The kanamycin (Kan)-resistance gene (1,473 bp) was amplified using primers Kan-R and Kan-F. The deletion-insertion mutant cassette was amplified with overlapping PCR using the upstream and downstream fragments and the Kan-resistance gene as the templates, with primers *lrgAB*-1F and *lrgAB*-2R. The *lrgAB* deletion-insertion mutant cassette was inserted into the *Bam*HI and *Eco*RI restriction sites of the pMAD plasmid to generate the recombinant plasmid pMAD-Δ*lrgAB*, which was then used to transform *Bt* HD73 cells with electroporation. Transformants were grown at 30°C in LB plate containing erythromycin and kanamycin, and then transferred to liquid LB containing kanamycin at 39°C. The cells were then plated on LB agar plates. Colonies with kanamycin resistance but lacking erythromycin resistance were selected, and one mutant strain, HDΔ*lrgAB*, was verified with PCR.

### Genetic complementation of the *lrgAB* and *lytSR* deletion mutants

The oligonucleotide primer pairs *lrgAB*hf-F/*lrgAB*hf-R and *lytSR*hf-F/*lytSR*hf-R were used to amplify the *lrgAB* gene with its own promoter P*lrgAB*, and the *lytSR* gene with its promoter P*lytSR*. The resultant fragments were digested with *Pst*I/*Bam*HI and *Sal*I/*Eco*RI, respectively, and then integrated into the shuttle vector pHT315 (Arantes and Lereclus, 1991) to generate pHT*lrgAB* and pHT*lytSR*, respectively. The genetically complemented mutant strains Δ*lrgAB*(*lrgAB*) and Δ*lytSR*(*lytSR*) were generated by introducing pHT*lrgAB* and pHT*lytSR* into HDΔ*lrgAB* and -HDΔ*lytSR*, respectively.

### Construction of *spoIID, spoIIM*, and *spoIIP* mutants

*spoIID* (HD73_5692), *spoIIM* (HD73_4392), and *spoIIP* (HD73_2232) mutants were constructed similar to *lrgAB* as described above, but using the primer pairs *spoIID*-1F/*spoIID*-1R, *spoIID*-2F/*spoIID*-2R, kanD-F/kanD-R, *spoIIM*-1F/*spoIIM*-1R, *spoIIM*-2F/*spoIIM*-2R, kanM-F/kanM-R, *spoIIP*-1F/*spoIIP*-1R, *spoIIP*-2F/*spoIIP*-2R, and kanP-F/kanP-R, respectively. The recombinant plasmids pMAD-Δ*spoIID*, pMAD-Δ*spoIIM*, and pMAD-Δ*spoIIP* were electroporated into *Bt* HD73 cells. Colonies with kanamycin resistance but lacking erythromycin resistance were selected, and mutant strains, HDΔ*spoIID*, HDΔ*spoIIM*, and HDΔ*spoIIP*, were verified with PCR.

### Growth curve assays

Overnight cultures of each strain grown in LB medium were used as starters for growth curve analyses. The exponential growth phase cells were washed in phosphate-buffered saline and then inoculated into SSM or M9 medium supplemented with tryptophan (50 μg/ml) and pyruvate (6 g/l) to an optical density at 600 nm (OD_600_) of 0.1. The cultures were incubated at 30°C with shaking at 220 rpm, and growth was monitored by measuring the absorbance at 600 nm at different timepoints. Values represent the means of at least three independent replicates. Error bars represent standard deviations.

### Determination of sporulation efficiency

The HD73, HDΔ*lrgAB*, Δ*lrgAB(lrgAB)*, HDΔ*lytSR*, and Δ*lytSR*(*lytSR*) strains were grown in SSM to *T*_28_ (*T*_0_ is the end of the exponential phase, and *T*n is n hours after *T*_0_) at 30°C with vigorous shaking. The number of viable cells was counted as the total colony-forming units (CFU) on the LB plates. The number of spores was determined as the number of heat-resistant (65°C for 30 min) CFU on the LB plates. Sporulation efficiency was defined as the ratio of the number of spores to the number of viable cells, multiplied by 100. Values represent the means of at least three independent replicates. The data were analyzed with SPSS (version 19.0) using a *t*-test. Error bars represent standard deviations. *P*-values are indicated in the figure legend.

### Laser scanning confocal microscopy

The vital membrane dye FM4-64 (Molecular Probes, Inc., Eugene, OR, USA) was dissolved in dimethyl sulfoxide to a final concentration of 100 μM. The cells were stained with FM4-64 (100 μM) for 1 min on ice (Yang J. et al., 2013). To assess engulfment, 0.5 ml of cells cultured to *T*_12_ were pelleted and resuspended in 0.1 ml of H_2_O. An aliquot (2 μl) of this cell suspension was placed on a slide and stained with FM4-64 (100 μM) and MitoTracker Green FM (MTG, 100 nM; from Molecular Probes) for 1 min, and then scanned (476–490 nm excitation and 510–667 nm emission) with a confocal laser scanning microscope (Leica TCS SL; Leica Microsystems, Wetzlar, Germany). Each strain was scanned independently at least three times and each scan was then viewed in at least five fields. The rate of incomplete engulfment was defined as the ratio of the number of incompletely engulfed cells (stained with FM4-64 in the mother cell) to the total number of cells. The values given are the means of at least three independent replicates.

### Construction of promoter fusions with *lacZ*

To assess the transcriptional activity of P*lrgAB* and P*lytSR* promoters, putative promoter fragments (633 and 845 bp, respectively) were cloned from *Bt* HD73 genomic DNA using the primer pairs P*lrgAB*-F/P*lrgAB*-R and P*lytSR*-F/P*lytSR*-R, respectively. The *Pst*I/*Bam*HI fragments of P*lrgAB* and P*lytSR* were separately integrated into vector pHT304-18Z, which is the *Bt-E. coli* shuttle harboring a promoterless *lacZ* gene (Agaisse and Lereclus, 1994) to generate plasmids pHT304P*lrgAB* and pHT304P*lytSR*, respectively. The former was introduced into *Bt* strain HD73 and the HDΔ*lytSR* mutant, whereas the latter was introduced into *Bt* strain HD73 and the HDΔ*sigE* mutant (Du et al., 2012). Resultant HD(P*lrgAB*), Δ*lytSR*(P*lrgAB*), HD(P*lytSR*), and Δ*sigE*(P*lytSR*) strains were selected with erythromycin and verified with PCR.

The constructions of P*spoIID*, P*spoIIM*, and P*spoIIP* (650, 437, and 668 bp, respectively) with *lacZ* fusions are similar to P*lrgAB* as described above, but using the primer pairs P*spoIID*-F/P*spoIID*-R, P*spoIIM*-F/P*spoIIM*-R, and P*spoIIP*-F/P*spoIIP*-R, respectively. The recombinant plasmids pHT304P*spoIID*, pHT304P*spoIIM*, and pHT304P*spoIIP* were introduced into *Bt* strain HD73 and the HDΔ*lytSR* mutant. The resultant strains Δ*lytSR*(P*spoIID*), Δ*lytSR*(P*spoIIM*), and Δ*lytSR*(P*spoIIP*) were selected with erythromycin and verified with PCR.

### β-galactosidase activity assay

*Bt* strains carrying *lacZ* transcriptional fusions were cultured in liquid SSM and 2-ml samples were collected at 1-h intervals. The cells were pelleted and resuspended in 0.5 ml of Z buffer (Peng et al., 2014) at 4°C, then lysed with a Mini-Beadbeater cell disrupter (BioSpec, Bartlesville, OK, USA) and centrifuged at 10,000 × *g* for 7 min at 4°C. β-Galactosidase activity was determined as previously described (Perchat et al., 2011). The reported values are the means of at least three independent assays. The data were analyzed with SPSS (version 19.0) using a *t*-test. Error bars represent standard deviations.

## Results

### PlrgAB promoter transcription is regulated by LytSR

**Figure 1:**
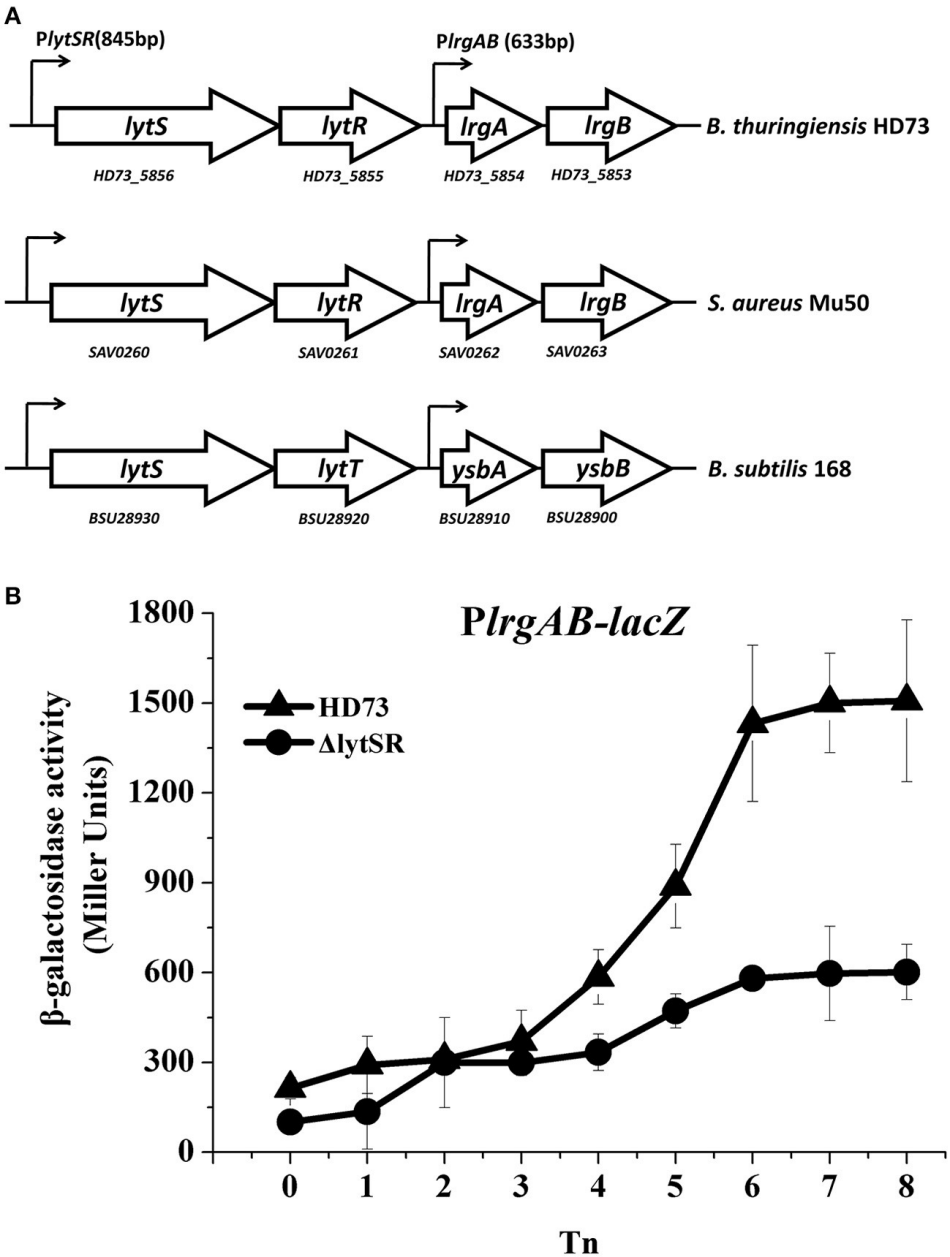
P*lrgAB* transcription in wild-type *Bt* HD73 and the *lytSR* mutant. **(A)** Gene organization at the *lytSR*–*lrgAB* locus in *Bt* HD73, *S. aureus* and *B. subtilis*. White arrows represent open reading frames (ORFs); small arrows denote the lengths of promoters upstream from the *lytS* and *lrgA* genes in *Bt*. **(B)** β-galactosidase activity from the *lrgAB* promoter (P*lrgAB*) in HD73 (▴) and *lytSR* mutant (•) grown in SSM. *T*_0_ is the end of the exponential phase; *T*n is n hours after *T*_0_. Values represent the means of at least three independent replicates; error bars represent standard deviations.

*lrgAB* is located downstream from the *lytSR* genes in *Bt* HD73 (Figure 1A). The *Bt lytS* (*HD73_5856*, sensor histidine kinase), *lytR* (*HD73_5855*, response regulator), *lrgA* (*HD73_5854*, holin-like protein), and *lrgB* (*HD73_5853*, holin-like protein) genes encode proteins that share 50, 44, 44, and 54% amino acid sequence identity, respectively, with homologs in *Staphylococcus aureus* (Patel and Golemi-Kotra, 2015), and 66, 65, 62, and 78% amino acid sequence identity with homologs in *B. subtilis* (van den Esker et al., 2017). Alignments of these proteins from *Bt, S. aureus*, and *B. subtilis* are shown in Supplementary Figure 1. The two-component system LytSR/LytST contained the conserved His_kinase domain and the response regulator receiver domain in *Bt, S. aureus*, and *B. subtilis* (Supplementary Figure 1).

To investigate the transcription from and regulation of the P*lrgAB* promoter in *Bt, Bt* strain HD73, and the *lytSR* mutant HDΔ*lytSR* were transformed with a P*lrgAB-lacZ* fusion construct. The results of the β-galactosidase assay showed that the transcriptional activity of P*lrgAB* increased from *T*_4_ to *T*_8_ in the HD73 strain in SSM, whereas it did not increase dramatically in the HDΔ*lytSR* mutant (Figure 1B), suggesting that the transcription of the *lrgAB* genes is positively regulated by LytSR during the late sporulation process.

### LytSR modulates *Bt* forespore engulfment

**Figure 2:**
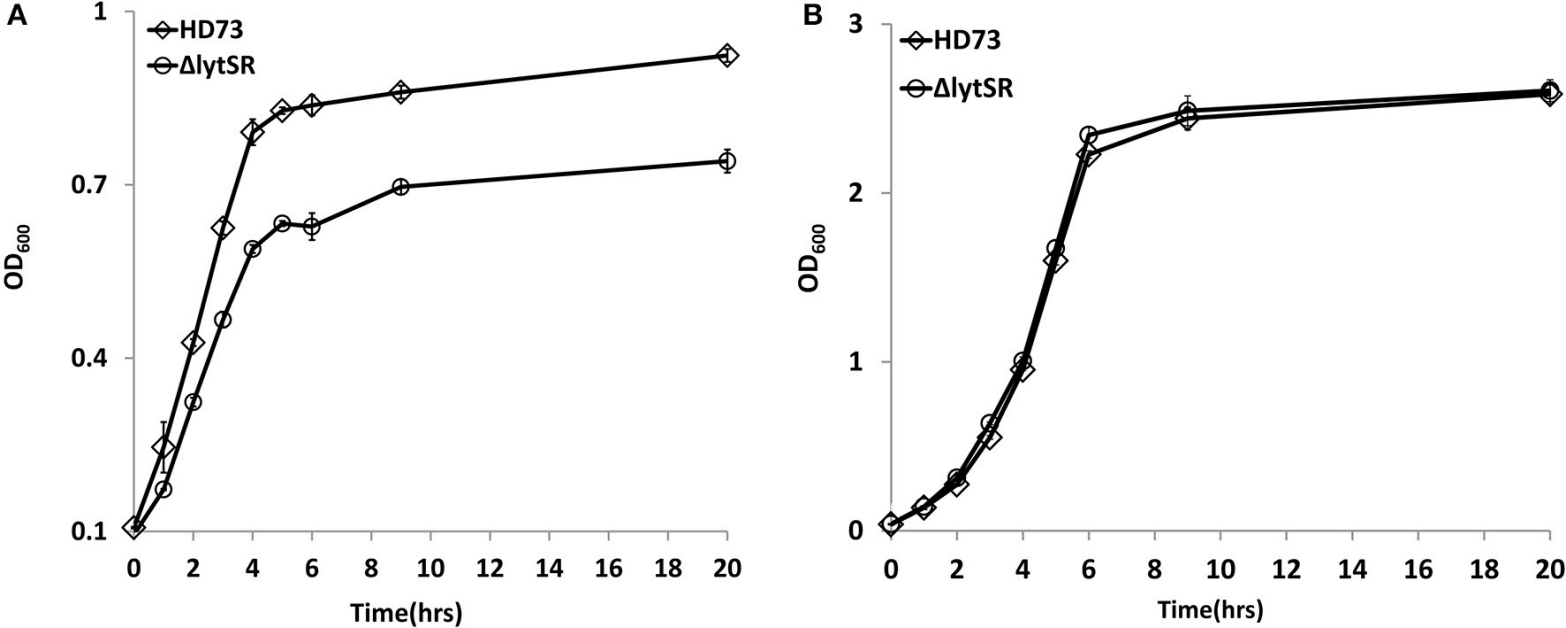
Growth curves assay. Wild-type HD73 (♢) and *lytSR* mutant cells (◦) were grown in M9 supplemented with pyruvate **(A)**, and SSM **(B)**. Values represent the means of at least three independent replicates; Error bars represent standard deviations.

Previous studies have shown that LytSR/LytST is involved in pyruvate utilization (Zhu et al., 2010; van den Esker et al., 2017). We also compared the growth of the *lytSR* mutant with that of wild-type strain HD73 in the presence of pyruvate. Results showed that Δ*lytSR* was unable to grow in M9 medium supplemented with pyruvate, whereas the wild-type reached an OD_600_ of 0.9 after 20 h of incubation (Figure 2A), suggesting that LytSR is involved in pyruvate utilization in *Bt*. However, no differences in the growth curves of Δ*lytSR* and the wild-type were observed in SSM (Figure 2B). Thus, in order to eliminate the effects of growth medium, we selected SSM for further analyses of the sporulation efficiency.

**Figure 3:**
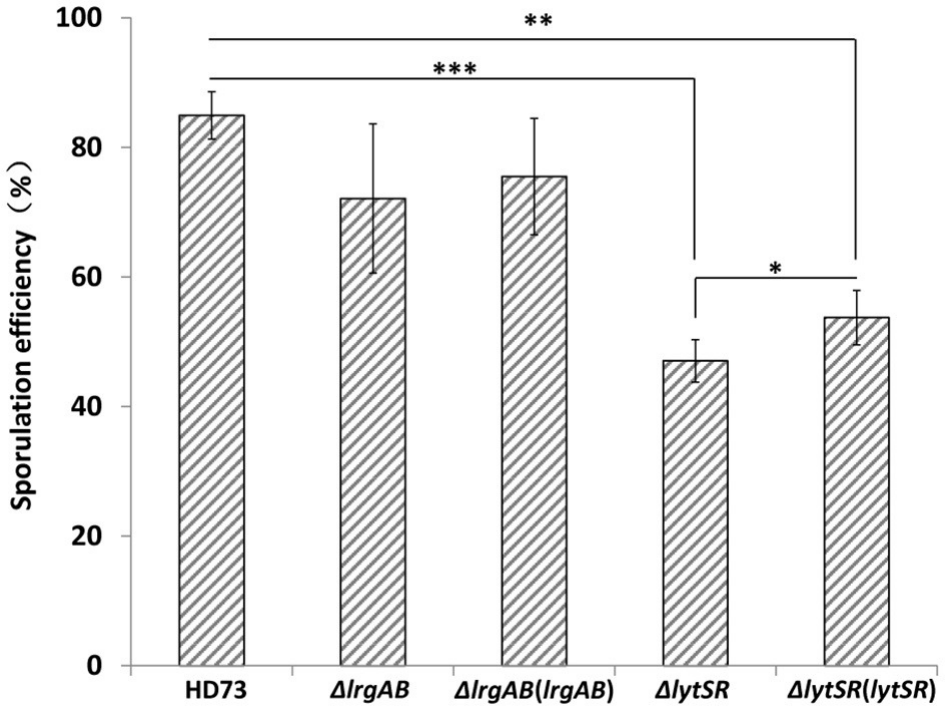
Analysis of sporulation efficiency. Sporulation efficiencies of wild-type HD73, Δ*lytSR*, Δ*lrgAB*, Δ*lrgAB*(*lrgAB*), and Δ*lytSR*(*lytSR*) were compared. Sporulation efficiency was defined as the ratio of the number of spores to the number of viable cells, multiplied by 100. Values represent the means of at least three independent replicates. The data were analyzed with SPSS (version 19.0) using a *t*-test. Error bars represent standard deviations. ^*^P ≤ 0.05; ^**^P ≤ 0.01; ^***^P ≤ 0.001.

Because the *lrgAB* genes have a dramatic impact on sporulation in *B. anthracis* (Chandramohan et al., 2009), we predicted that the *lytSR* or *lrgAB* mutation would affect the ability of the *Bt* cells to undergo sporulation. Therefore, the abilities of the *lrgAB* and *lytSR* mutants to sporulate were assessed. The wild-type strain HD73 had a sporulation efficiency of 85 ± 4% after growth to *T*_28_ in SSM (Figure 3). The sporulation efficiency was not significantly different between HD73 and either HDΔ*lrgAB* (72 ± 11%) or Δ*lrgAB*(*lrgAB*) (76 ± 9%), whereas it was significantly reduced in both HDΔ*lytSR* (47 ± 3%, *P* ≤ 0.001) and, the genetically complemented strain Δ*lytSR*(*lytSR*) (54 ±4%, *P* ≤ 0.01). Based on the *P*-values (*P* ≤ 0.05, Figure 3) between Δ*lytSR* and Δ*lytSR*(*lytSR*), Δ*lytSR*(*lytSR*) showed a partly restored sporulation function. These results indicate that *lytSR* affects spore formation and the regulation of the genes involved in sporulation.

**Figure 4:**
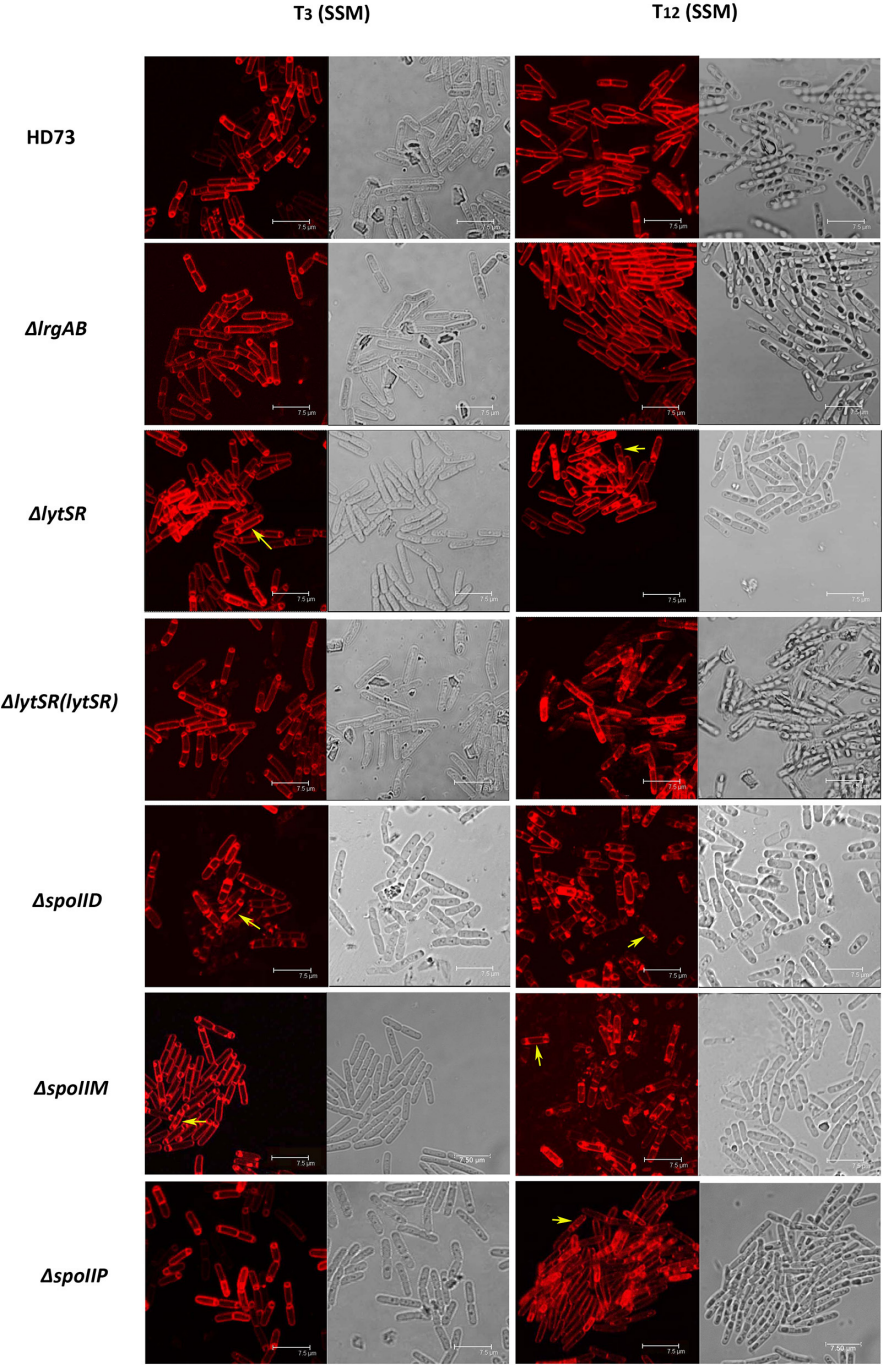
Sporulation process in *Bt* HD73 in SSM. Laser scanning confocal micrographs of *Bt* wild-type HD73 cells and Δ*lrgAB*, Δ*lytSR*, Δ*lytSR*(*lytSR*), Δ*spoIID*, Δ*spoIIM, and* Δ*spoIIP* cells grown in SSM to *T*_3_ and *T*_12_ (30°C). Cell membrane is visible as red fluorescence. Yellow arrow indicates a bipolar septum. Bar, 7.5 μm.

**Figure 5:**
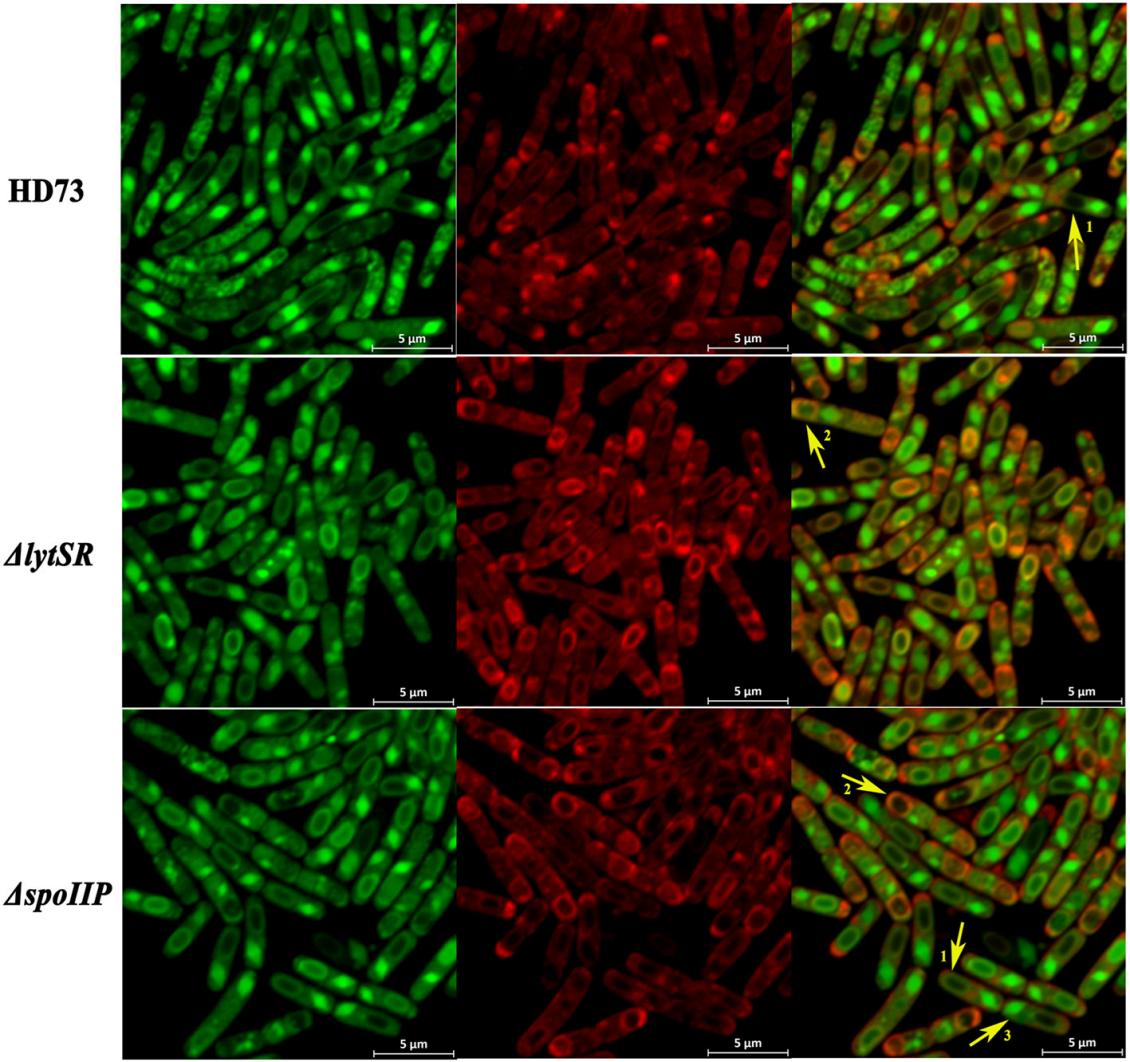
Schematic representation of the membrane fusion assay. Laser scanning confocal micrographs of *Bt* wild-type HD73, Δ*lytSR, and* Δ*spoIIP* cells grown in SSM to *T*_12_ (30°C). Red lines represent membranes stained with FM4-64 and MitoTracker Green FM (MTG), and green lines indicate membranes stained with MTG only. Arrow 1 points to cells that have completed the process of engulfment; only the mother–cell membranes are stained with FM4-64, but MTG stained both the forespore and mother–cell membranes. Arrow 2 points to cells that have undergone incomplete engulfment, and the membrane fusion is stained with FM4-64 and MTG. Arrow 3 points to the crystal protein stained with MTG only.

To determine the effect of LytSR on sporulation in *Bt* HD73, the cell membranes of *Bt* HD73 and its mutants were stained with the vital dye FM4-64, which labels the plasma membranes of living cells, and the process of spore formation was visualized with confocal microscopy. In cells grown to *T*_3_ in SSM, the polar septum was curved in the wild-type and mutant cells, whereas some cells of HDΔ*lytSR* had an incomplete septum at the distal pole (Figure 4). At *T*_12_, the process of engulfment was completed in the forespores of the wild-type (Figure 5, arrow 1) and HDΔ*lrgAB* cells. In these cases, the spores were not labeled with FM4-64, but were stained with MTG, and only the outer membranous outline of the living cells could be observed. In the mutant HDΔ*lytSR*, a proportion of the cells had completed the process of engulfment, but 52 ± 3% cells were arrested in forespore engulfment (Figure 5, arrow 2), and a bipolar septum phenotype was observed.

### *lytSR* transcription is controlled by SigE

**Figure 6:**
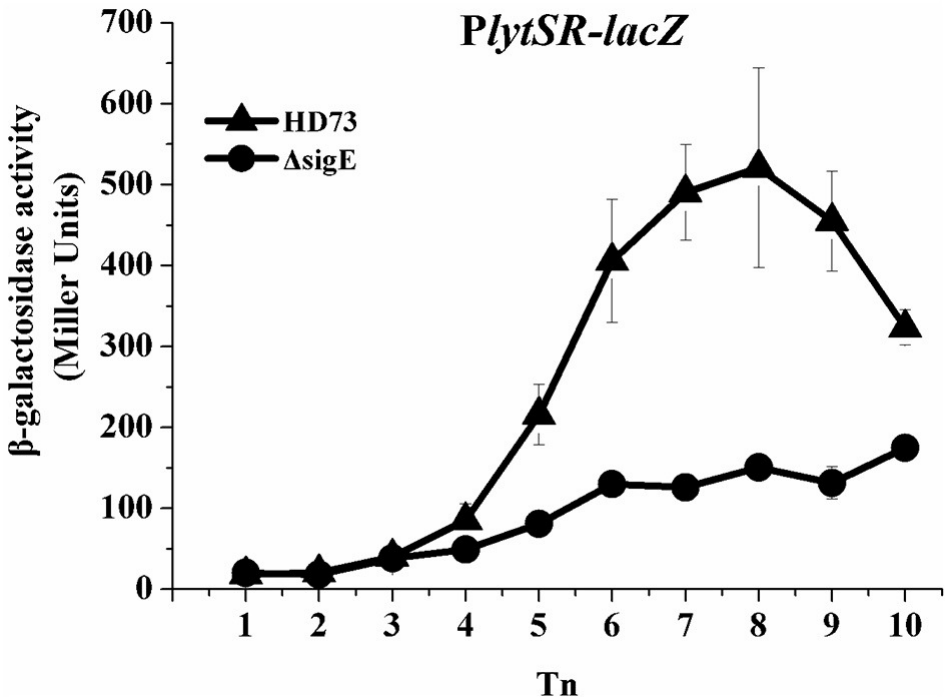
Transcription of P*lytSR* promoter in *Bt*. Wild-type HD73 (▴) and *sigE* mutant cells (•) were grown in SSM. *T*_0_ is the end of the exponential phase, and *T*_n_ is n hours after *T*_0_. Values represent the means of at least three independent replicates; error bars represent standard deviations.

HDΔ*lytSR* cells were unable to initiate engulfment or form bipolar septa. The mother–cell-specific sigma factor SigE plays a critical role in the formation of an asymmetric septum and in forespore engulfment (Errington, 2003). Therefore, we predicted that SigE would also affect the transcription of *lytSR*. The results of the β-galactosidase assay indicate that the transcriptional activity of P*lytSR* increased rapidly from *T*_4_ to *T*_10_ in wild-type HD73, whereas it increased much more slowly in the HDΔ*sigE* mutant grown in SSM (Figure 6), suggesting that the transcription of *lytSR* is controlled by the mother-cell-specific sigma factor SigE.

### LytSR affects *spoIIP* expression

**Figure 7:**
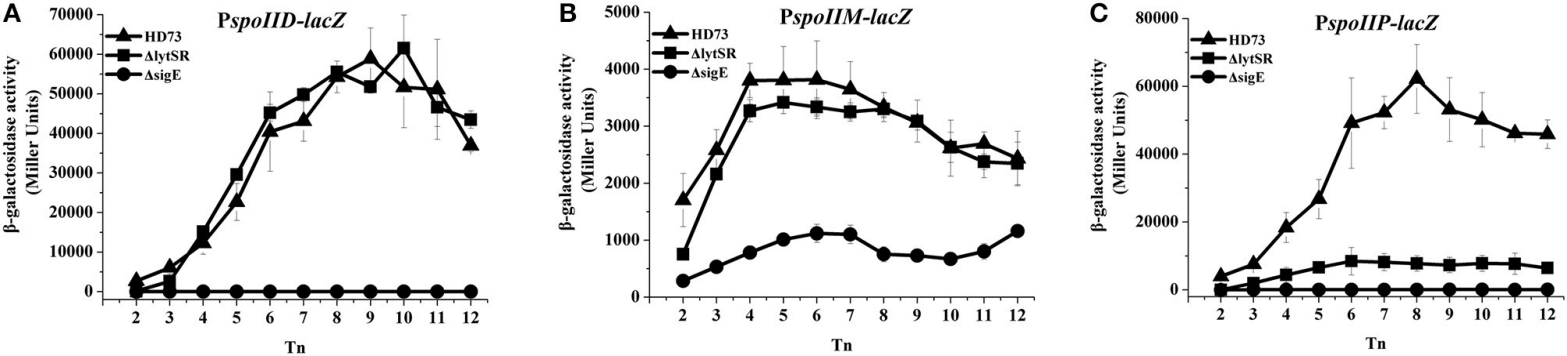
Transcription of P*spoIID*, P*spoIIM*, and P*spoIIP* promoters in *Bt*. Transcription of P*spoIID* **(A)**, P*spoIIM* **(B)**, and P*spoIIP* **(C)** in wild-type HD73 (▴), *lytSR* mutant (■), and *sigE* mutant (•) cells grown in SSM. *T*_0_ is the end of the exponential phase, and *T*_n_ is n hours after *T*_0_. Values represent the means of at least three independent replicates; error bars represent standard deviations.

In *B. subtilis*, the sporulation genes *spoIID, spoIIM*, and *spoIIP* are controlled by SigE (Eichenberger et al., 2001) and may also be involved in suppressing septum formation at the distal pole of the sporangium (Chastanet and Losick, 2007). To determine whether LytSR affects the process of spore engulfment by regulating the expression of *spoIID, spoIIM*, and *spoIIP*, the promoters of these genes were fused to *lacZ* and the β-galactosidase activity was assessed in wild-type HD73 cells, *lytSR* and *sigE* mutants. The results showed that the transcriptional activities of *spoIID, spoIIM*, and *spoIIP* were sharply reduced or abolished in the *sigE* mutant grown in SSM (Figure 7), suggesting that the transcription of *spoIID, spoIIM*, and *spoIIP* is directly controlled by SigE in *Bt*. The transcription of P*spoIID* and P*spoIIM* transcription did not differ between the wild-type and mutants grown in SSM (Figures 7A,B). However, P*spoIIP* activity was dramatically reduced in the *lytSR* mutant grown in SSM (Figure 7C). These results suggest that the transcription of *spoIIP* is affected by LytSR.

### LytSR mainly modulates *Bt* forespore engulfment by regulating *spoIIP* expression

To determine whether LytSR modulates *Bt* forespore engulfment by regulating *spoIIP* expression, we observed the phenotypes of the *spoIID, spoIIM*, and *spoIIP* mutants in SSM. In cells grown to *T*_3_ or *T*_12_ in SSM, the polar septum was curved or had completed the process of engulfment in the wild-type, whereas some HDΔ*spoIIM*, and HDΔ*spoIID* cells displayed an incomplete septum at the distal pole (Figure 4). At *T*_12_, the phenotype of HDΔ*spoIIP* was similar to that of HDΔ*lytSR*, and the only difference was that more HDΔ*spoIIP* cells (68 ± 5%) than HDΔ*lytSR* cells (48 ± 3%) had completed the process of engulfment (Figure 5). In contrast, almost all the HDΔ*spoIID* and HDΔ*spoIIM* cells arrested in forespore engulfment, and bipolar septa were also observed (Figure 4), so this phenotype is similar to that of the *spoIID* and *spoIIM* mutants of *B. subtilis* (Pogliano et al., 1999). The β-galactosidase activity assay also revealed that P*spoIIP* was dramatically reduced in the *lytSR* mutant grown in SSM (Figure 7). All these results indicate that LytSR modulates *Bt* forespore engulfment, mainly by affecting *spoIIP* expression.

## Discussion

The sporulation efficiency assay and a confocal microscopic analysis showed that spore formation was unaffected in the *Bt lrgAB* mutant. This differs from the dramatic impact of this mutation on sporulation efficiency observed in *B. anthracis* (Chandramohan et al., 2009), although orthologues of the *lrgAB* locus of *Bt* HD73 are conserved in the genomes of the *B. cereus* group (Supplementary Figure 2). These genes share high sequence similarity and a similar organization with those of the *lrgAB* locus. However, in the *Bt lytSR* mutant, sporulation efficiency was markedly reduced and spore engulfment was lower than wild-type, and a bipolar septum was observed in some cells grown in SSM. These results indicate that LytSR does not modulate the process of spore formation by regulating of *lrgAB*, but probably by controlling the expression of other genes.

LytSR and LrgAB are widely conserved in both the *B. cereus* group and amongst other bacterial species (Supplementary Figure 2). In *S. aureus*, the LytSR are involved in the regulation of bacterial programmed cell death, biofilm formation, and adaptation to cationic antimicrobial peptides (Brunskill and Bayles, 1996; Rice et al., 2005; Sharma-Kuinkel et al., 2009; Yang S. J. et al., 2013; Lehman et al., 2015), while in *Staphylococcus epidermidis*, they play a role in regulating extracellular murein hydrolase activity, bacterial cell death, and pyruvate utilization (Zhu et al., 2010). In *B. subtilis*, the *lytSR* and *lrgA* homologs *lytST* and *ysbA* are not involved in programmed cell death, but are essential for pyruvate transport or utilization (van den Esker et al., 2017). We also found that mutation of *lytSR* has an effect on pyruvate utilization in M9 medium in *Bt* (Figure 2A). However, no differences in the growth curves of Δ*lytSR* and the wild-type were observed in SSM (Figure 2B). We further demonstrated that the LytSR are involved in the process of spore engulfment in *Bt* in SSM. These results indicate that LytSR does not modulate the process of spore formation by affecting the pyruvate utilization.

A high proportion (61%) of *B. subtilis sigE* mutant cells had complete septa near both the poles and failed to undergo engulfment. SigE direct controls the transcription of the sporulation genes *spoIID, spoIIM*, and *spoIIP* (Eichenberger et al., 2001). Single mutants of these genes prevent engulfment as they are defective in the dissolution of the peptidoglycan layer between the two membranes of the polar septum. Instead, the septal membrane bulges through the incompletely degraded cell wall layer. In double mutants, the bulge is less prominent, and only in the absence of all three proteins does septum formation occur at both poles at a frequency similar to that observed in the *sigE* mutant (Eichenberger et al., 2001; Meyer et al., 2010; Tan and Ramamurthi, 2014). The transcription of *spoIID, spoIIM*, and *spoIIP* is controlled by SigE in *Bt* (Figure 7) as in *B. subtilis*, and the transcriptional activity of *spoIIP* was sharply reduced in the *lytSR* mutant compared with that in the wild-type strain grown in SSM. However, the transcriptional activities of *spoIIM* and *spoIID* in *lytSR* mutant did not differ from those in the wild-type strain. This observation suggests that the effect of *lytSR* on *spoIIP* expression does not result from the direct activity of LytR on the transcription of *spoIIP*. The effect of the *lytSR* mutation on *spoIIP* expression might be attributable to the low availability of active SigE in the mother–cell compartment of the mutant strain. The transcription of *spoIIP* requires SigE. However, the amount of SigE required for the full expression of *spoIIM, spoIID*, and *spoIIP* might differ, as has been demonstrated for the genes of the Spo0A regulon, which are distributed in two classes: those that are regulated at a low dose of Spo0A-P and those that require a high dose to be activated or repressed (Fujita et al., 2005). In a similar way, *spoIIP* transcription might require larger amounts of SigE than the transcription of *spoIIM* and *spoIID*. Therefore, the SigE defect in the *lytSR* mutant would have a more dramatic effect on *spoIIP* expression than on *spoIIM* or *spoIID* expression.

The transcriptional analysis of *lytSR* in the *sigE* mutant and of *spoIID, spoIIM*, and *spoIIP* in the *lytSR* mutant revealed that the *lytSR* operon is controlled by SigE and that the efficacy of *spoIIP* transcription depends, directly or indirectly, on *lytSR*. We have demonstrated that LytSR affects spore formation by preventing the correct engulfment of the forespore. However, we did not determine whether this effect is responsible for the defect in *spoIIP* expression or, reciprocally, if it is caused by weak *spoIIP* expression. In *B. subtilis*, SpoIIP is targeted to the septal membrane by SpoIIM, where it interacts with SpoIID, which also localizes to the membrane via its interaction with SpoIIP. SpoIIP and SpoIID have complementary enzymatic activities, which are similar to those of LytB and LytC (CwlB), respectively, the major vegetative autolysins involved in peptidoglycan degradation (Shida et al., 2001; Chastanet and Losick, 2007; Gutierrez et al., 2010). Therefore, we infer that LytSR modulates spore engulfment by directly or indirectly inducing the transcription of the sporulation gene *spoIIP*. However, many other genes are also involved in engulfment and must be examined in future studies because they may be more directly responsible for the sporulation phenotype.

## Author contributions

FS designed the research. QP and JW performed the experimental work. QP drafted the manuscript. JW, XC, and LQ constructed the mutants, analyzed the sporulation efficiency and perform the laser scanning confocal microscopy. FS, JZ, and HT critically revised the manuscript for intellectual content. All authors read and approved the final version of the manuscript.

### Conflict of interest statement

The authors declare that the research was conducted in the absence of any commercial or financial relationships that could be construed as a potential conflict of interest.

## References

[B1] Agaisse H. Lereclus D. (1994). Structural and functional analysis of the promoter region involved in full expression of the cryIIIA toxin gene of *Bacillus thuringiensis*. Mol. Microbiol. 13, 97–107. 10.1111/j.1365-2958.1994.tb00405.x 7984098

[B2] Arantes O. Lereclus D. (1991). Construction of cloning vectors for *Bacillus-thuringiensis*. Gene 108, 115–120. 10.1016/0378-1119(91)90495-W 1662180

[B3] Arnaud M. Chastanet A. Débarbouillé M. (2004). New vector for efficient allelic replacement in naturally nontransformable, low-GC-content, gram-positive bacteria. Appl. Environ. Microbiol. 70, 6887–6891. 10.1128/AEM.70.11.6887-6891.2004 15528558 PMC525206

[B4] Stenfors Arnesen L. P. Fagerlund A. Granum P. E. (2008). From soil to gut: *Bacillus cereus* and its food poisoning toxins. FEMS Microbiol. Rev. 32, 579–606. 10.1111/j.1574-6976.2008.00112.x 18422617

[B5] Brunskill E. W. Bayles K. W. (1996). Identification and molecular characterization of a putative regulatory locus that affects autolysis in *Staphylococcus aureus*. J. Bacteriol. 178, 611–618. 10.1128/jb.178.3.611-618.1996 8550490 PMC177702

[B6] Burbulys D. Trach K. A. Hoch J. A. (1991). Initiation of sporulation in *B. subtilis* is controlled by a multicomponent phosphorelay. Cell 64, 545–552. 10.1016/0092-8674(91)90238-T 1846779

[B7] Chandramohan L. Ahn J.-S. Weaver K. E. Bayles K. W. (2009). An overlap between the control of programmed cell death in *Bacillus anthracis* and sporulation. J. Bacteriol. 191, 4103–4110. 10.1128/JB.00314-09 19411321 PMC2698511

[B8] Chastanet A. Losick R. (2007). Engulfment during sporulation in *Bacillus subtilis* is governed by a multi-protein complex containing tandemly acting autolysins. Mol. Microbiol. 64, 139–152. 10.1111/j.1365-2958.2007.05652.x 17376078

[B9] de Been M. Tempelaars M. H. Van Schaik W. Moezelaar R. Siezen R. J. Abee T. (2010). A novel hybrid kinase is essential for regulating the sigma B-mediated stress response of *Bacillus cereus*. Environ. Microbiol. 12, 730–744. 10.1111/j.1462-2920.2009.02116.x 19958380

[B10] Du L. Qiu L. Peng Q. Lereclus D. Zhang J. Song F.. (2012). Identification of the promoter in the intergenic region between orf1 and cry8Ea1 controlled by sigma H factor. Appl. Environ. Microbiol. 78, 4164–4168. 10.1128/AEM.00622-12 22504821 PMC3370531

[B11] Eichenberger P. Fawcett P. Losick R. (2001). A three-protein inhibitor of polar septation during sporulation in *Bacillus subtilis*. Mol. Microbiol. 42, 1147–1162. 10.1046/j.1365-2958.2001.02660.x 11886548

[B12] Errington J. (2003). Regulation of endospore formation in *Bacillus subtilis*. Nat. Rev. Microbiol. 1, 117–126. 10.1038/nrmicro750 15035041

[B13] Fujita M. Gonzalez-Pastor J. E. Losick R. (2005). High- and low-threshold genes in the Spo0A regulon of *Bacillus subtilis*. J. Bacteriol. 187, 1357–1368. 10.1128/JB.187.4.1357-1368.2005 15687200 PMC545642

[B14] Fujita M. Losick R. (2003). The master regulator for entry into sporulation in *Bacillus subtilis* becomes a cell-specific transcription factor after asymmetric division. Genes Dev. 17, 1166–1174. 10.1101/gad.1078303 12730135 PMC196045

[B15] Gutierrez J. Smith R. Pogliano K. (2010). SpoIID-mediated peptidoglycan degradation is required throughout engulfment during *Bacillus subtilis* sporulation. J. Bacteriol. 192, 3174–3186. 10.1128/JB.00127-10 20382772 PMC2901704

[B16] Hilbert D. W. Piggot P. J. (2004). Compartmentalization of gene expression during *Bacillus subtilis* spore formation. Microbiol. Mol. Biol. Rev. 68, 234–262. 10.1128/MMBR.68.2.234-262.2004 15187183 PMC419919

[B17] Jiang M. Shao W. L. Perego M. Hoch J. A. (2000). Multiple histidine kinases regulate entry into stationary phase and sporulation in *Bacillus subtilis*. Mol. Microbiol. 38, 535–542. 10.1046/j.1365-2958.2000.02148.x 11069677

[B18] Lehman M. K. Bose J. L. Sharma-Kuinkel B. K. Moormeier D. E. Endres J. L. Sadykov M. R.. (2015). Identification of the amino acids essential for LytSR-mediated signal transduction in *Staphylococcus aureus* and their roles in biofilm-specific gene expression. Mol. Microbiol. 95, 723–737. 10.1111/mmi.12902 25491472 PMC4347461

[B19] Lereclus D. Arantes O. Chaufaux J. Lecadet M. (1989). Transformation and expression of a cloned delta-endotoxin gene in *Bacillus thuringiensis*. FEMS Microbiol. Lett. 51, 211–217. 2550317 10.1016/0378-1097(89)90511-9

[B20] Liu G. Song L. Shu C. Wang P. Deng C. Peng Q.. (2013). Complete genome sequence of *Bacillus thuringiensis* subsp. kurstaki strain HD73. Genome Announc. 1:e00080–13. 10.1128/genomeA.00080-13 23516207 PMC3622971

[B21] Meyer P. Gutierrez J. Pogliano K. Dworkin J. (2010). Cell wall synthesis is necessary for membrane dynamics during sporulation of *Bacillus subtilis*. Mol. Microbiol. 76, 956–970. 10.1111/j.1365-2958.2010.07155.x 20444098 PMC2893020

[B22] Ohara K. Fukuda T. Okada H. Kitao S. Ishida Y. Kato K.. (2015). Identification of significant amino acids in multiple transmembrane domains of human transient receptor potential ankyrin 1 (TRPA1) for activation by eudesmol, an oxygenized sesquiterpene in hop essential oil. J. Biol. Chem. 290, 3161–3171. 10.1074/jbc.M114.600932 25525269 PMC4317010

[B23] Patel K. Golemi-Kotra D. (2015). Signaling mechanism by the *Staphylococcus aureus* two-component system LytSR: role of acetyl phosphate in bypassing the cell membrane electrical potential sensor LytS. F1000Res. 4:79. 10.12688/f1000research.6213.1 27127614 PMC4830213

[B24] Peng Q. Yang M. Wang W. Han L. Wang G. Wang P.. (2014). Activation of gab cluster transcription in *Bacillus thuringiensis* by gamma-aminobutyric acid or succinic semialdehyde is mediated by the Sigma 54-dependent transcriptional activator GabR. BMC Microbiol. 14:306. 10.1186/s12866-014-0306-3 25527261 PMC4279683

[B25] Perchat S. Dubois T. Zouhir S. Gominet M. Poncet S. Lemy C.. (2011). A cell-cell communication system regulates protease production during sporulation in bacteria of the *Bacillus cereus* group. Mol. Microbiol. 82, 619–633. 10.1111/j.1365-2958.2011.07839.x 21958299

[B26] Pogliano J. Osborne N. Sharp M. D. Abanes-De Mello A. Perez A. Sun Y. L.. (1999). A vital stain for studying membrane dynamics in bacteria: a novel mechanism controlling septation during *Bacillus subtilis* sporulation. Mol. Microbiol. 31, 1149–1159. 10.1046/j.1365-2958.1999.01255.x 10096082 PMC2885269

[B27] Rice K. C. Nelson J. B. Patton T. G. Yang S. J. Bayles K. W. (2005). Acetic acid induces expression of the *Staphylococcus aureus* cidABC and lrgAB murein hydrolase regulator operons. J. Bacteriol. 187, 813–821. 10.1128/JB.187.3.813-821.2005 15659658 PMC545714

[B28] Schaeffer P. Millet J. Aubert J.-P. (1965). Catabolic repression of bacterial sporulation. Proc. Natl. Acad. Sci. U.S.A. 54, 704–711. 10.1073/pnas.54.3.704 4956288 PMC219731

[B29] Schnepf E. Crickmore N. Van Rie J. Lereclus D. Baum J. Feitelson J. (1998). *Bacillus thuringiensis* and its pesticidal crystal proteins. Microbiol. Mol. Biol. Rev. 62, 775–806. 9729609 10.1128/mmbr.62.3.775-806.1998 PMC98934

[B30] Sharma-Kuinkel B. K. Mann E. E. Ahn J. S. Kuechenmeister L. J. Dunman P. M. Bayles K. W. (2009). The *Staphylococcus aureus* LytSR two-component regulatory system affects biofilm formation. J. Bacteriol. 191, 4767–4775. 10.1128/JB.00348-09 19502411 PMC2715716

[B31] Shida T. Hattori H. Ise F. Sekiguchi J. (2001). Mutational analysis of catalytic sites of the cell wall lytic N-acetylmuramoyl-L-alanine amidases Cw1C and Cw1V. J. Biol. Chem. 276, 28140–28146. 10.1074/jbc.M103903200 11375403

[B32] Skerker J. M. Prasol M. S. Perchuk B. S. Biondi E. G. Laub M. T. (2005). Two-component signal transduction pathways regulating growth and cell cycle progression in a bacterium: a system-level analysis. PLoS Biol. 3:e334. 10.1371/journal.pbio.0030334 16176121 PMC1233412

[B33] Song F. Peng Q. Brillard J. Buisson C. de Been M. Abee T.. (2012). A multicomponent sugar phosphate sensor system specifically induced in *Bacillus cereus* during infection of the insect gut. FASEB J. 26, 3336–3350. 10.1096/fj.11-197681 22611084

[B34] Tan I. S. Ramamurthi K. S. (2014). Spore formation in *Bacillus subtilis*. Environ. Microbiol. Rep. 6, 212–225. 10.1111/1758-2229.12130 24983526 PMC4078662

[B35] van den Esker M. H. Kovacs A. T. Kuipers O. P. (2017). YsbA and LytST are essential for pyruvate utilization in *Bacillus subtilis*. Environ. Microbiol. 19, 83–94. 10.1111/1462-2920.13454 27422364

[B36] van Schaik W. Tempelaars M. H. Zwietering M. H. de Vos W. M. Abee T. (2005). Analysis of the role of RsbV, RsbW, and RsbY in regulating sigma(B) activity in *Bacillus cereus*. J. Bacteriol. 187, 5846–5851. 10.1128/JB.187.16.5846-5851.2005 16077134 PMC1196065

[B37] Yang J. Peng Q. Chen Z. Deng C. Shu C. Zhang J.. (2013). Transcriptional regulation and characteristics of a novel N-Acetylmuramoyl-l-alanine amidase gene involved in *Bacillus thuringiensis* mother cell lysis. J. Bacteriol. 195, 2887–2897. 10.1128/JB.00112-13 23603740 PMC3697243

[B38] Yang S. J. Xiong Y. Q. Yeaman M. R. Bayles K. W. Abdelhady W. Bayer A. S. (2013). Role of the LytSR two-component regulatory system in adaptation to cationic antimicrobial peptides in *Staphylococcus aureus*. Antimicrob. Agents Chemother. 57, 3875–3882. 10.1128/AAC.00412-13 23733465 PMC3719743

[B39] Zhu T. Lou Q. Wu Y. Hu J. Yu F. Qu D. (2010). Impact of the *Staphylococcus epidermidis* LytSR two-component regulatory system on murein hydrolase activity, pyruvate utilization and global transcriptional profile. BMC Microbiol. 10:287. 10.1186/1471-2180-10-287 21073699 PMC2996381

