# Supplementary material for: Disruption of Two-component System LytSR Affects Forespore Engulfment in Bacillus thuringiensis
Source: Front Cell Infect Microbiol. 2017 Nov 3;7:468. doi: 10.3389/fcimb.2017.00468 (PMC5675857; doi:10.3389/fcimb.2017.00468)
Supplement: Supplementary file 1 [file Image1.PDF]

Supplementary Material

Disruption of Two-component System LytSR Affects Forespore Engulfment in *Bacillus thuringiensis*

Qi Peng[+], Jianbo Wu[+], Xiaomin Chen, Lili Qiu, Jie Zhang, Hongtao Tian[*], Fuping Song[*]

*** Correspondence:** Corresponding Author: Hongtao Tian (tht631022@163.com), Fuping Song (fpsong@ippcaas.cn).

1 Supplementary Figure 1

LytS:

```
HD73_5856   .............................................................MNENWLQG.....VSSSS     13
SAV0260     MLSLTMLLLERVGLIIILAYVLMNIPYFKNLMNRRRTWKARWQLCIIFSLFALMSNLTGIVIDHQHSLSGSVYFRLDDDV     80
BSU28930    MIHLMIMMLERVGIIVILGFILAHTKLFRQALQNQDGYKGKAILISIFSLFSIISNYTGIEIQRNMIVNNDWVFTIDPSG     80

HD73_5856   TIANTRIMGVGISGLLGGPIVGVGVGFIAGIHRYMLGGTTALSCAISSILAGIITGYIGYIFKKYNRTITPKFSAVLSVF     93
SAV0260     SLANTRVLTIGVAGLVGGPFVGLFVGVISGIFRVYMGGADAQVYLISSIFIGIIAGYFGLQAQRRKRYPSIAKSAMIGIV    160
BSU28930    SIANTRILGVEIGGLLGGPFVGAGIGILAGLHRFSLGGSTALSCAVSSILAGVLAGLIGRYFTKRYRMPTPRIAALVGIG    160

HD73_5856   IVSLEMLMILLIIED...GISVVKTIAIPMILVNSFGSFILLSMIQAILRQEENAKALQTHKVLRIADKTLPYFRQGLTE    170
SAV0260     MEMIQMLSILTFSHDKAYAVDLISLIALPMIIVNSVGTAIFMSIIISTLKQEEQMKAVQTHDVLQLMNQTLPYFKEGLNR    240
BSU28930    MESLQMIIILLMAKPFSDAWELVSMIGIPMILINGTGSFIFLSIIQAIIRKEEQARALETHRVLTIADQTLPFFRQGLNE    240

HD73_5856   ESCKHVAQIIHRFTGTDAVSLTDTEKILAHVGLASDHHIPSHSLITGLSKEVLNTGKIMKAKSREVINCQHEGCPLQAAI    250
SAV0260     ESAQQIAMIIKNLMKVSAVAITSKNEILSHVGAGSDHHIPTNEILTSLSKDVLKSGKLKEVHTKEEIGCSHPNCPLRAAI    320
BSU28930    NSCKSVAAIIHKLTGTDAVSLTDKEKILAHVGAGMDHHIPSKSLITGLSKKVIKTGHIMKAISQEEIECTHAECPLHAAI    320

HD73_5856   VIPLTSHGNTIGTLKLYFKNPNQLSRVEEELAEGLAKIFSTQLELGEAELQSKLLQDAEIKALQAQINPHFLFNAINTVS    330
SAV0260     VIPLEMHGSIVGTLKMYFTNPNDLTFVERQLAEGLANIFSSQIELGEAETQSKLLKDAEIKSLQAQVSPHFFFNSINTIS    400
BSU28930    VLPLTSNGNTIGTLKMYFKSPAGLSQVEEELAEGLAMLFSTQLELGEAELQSKLLKDAEIKALQAQVNPHFLFNAINTIS    400
                                                                    His_kinase domain
HD73_5856   ALCRTDVEKARKLLLQLSVYFRCNLQGARQLLIPLEQELNHVQAYLSLEQARFPNKYEVKMYIEDELKTTLVPPFVLQLL    410
SAV0260     ALVRINSEKARELLLELSYFFRANLQGSKQHTITLDKELSQVRAYLSLEQARYPGRFNININVEDKYRDVLVPPFLIQIL    480
BSU28930    ALCRTDVEKTRKLLLQLSVYFRSNLQGARQLLIPLSKELNHLNAYLSLEQARFPGKYKIELNIDSRLEQIEIPPFVLQVL    480

HD73_5856   VENALRHAFPKKQPVCEVEVHVFEKEG.MVHFEVKDNGQGIEEERLEQLGKMVVSSKKGTGTALYNINERLIGLFGKETM    489
SAV0260     VENAIKHAFTNRKQGNDIDVSVIKETATHVRIIVQDNGQGISKDKMHLLGETSVESESGTGSALENLNLRLKGLFGKSAA    560
BSU28930    VENALRHAFPKKQDICKVTVCVLSDDA.SVYMKVADNGRGIPPDVLPELGKKPFPSKEGTGTALYNLNQRLIGLFGQQAA    559

HD73_5856   LHIESELNKGTEITFIIPKKVGEE.EPGVKSIS                                                  521
SAV0260     LQFESTS.SGTTFWCVLPYERQEE.E.......                                                  584
BSU28930    LHISSEVHKGTEVSFQVPMQQMKEGEEHAQGVN                                                  592
```

LytR/LytT:

```
HD73_5855    MLKVLVVDDEMLARDELKYLLERTKEVEIIGEADCVEDALEELMQSRPDIVFLDIQLSDDNGFEIANILKKMKNPPAIVF    80
SAV0261      .MKALIIDDEPLARNELTYLLNEIGGFEEINEAENVKETLEALLINQYDIIFLDVNLMDENGIELGAKIQKMKEPPAIIF    79
BSU28920     MLRVLIVDDEMLARDELAYLLKRTNDEMEINEAENIESAFDQMMDQKPDLLFLDVDLSGENGFDIAKRLKKMKHPPAIVF    80
```

Response regulator receiver domain

```
HD73_5855    ATAYDQYALQAFEVDALDYILKPFDEERIVQTLKKYKKQKQAQIETKHEIKGTDVTAEMHKLALPIEESIVLVNIEDIIY    160
SAV0261      ATAHDQYAVQAFELNATDYILKPFGQKRIEQAVNKVRATKAKDDNNASAIANDMSANFDQSLPVEIDDKIHMLKQQNIIG    159
BSU28920     ATAYDQYALKAFEVDALDYLTKPFDEERIQQTLKKYKKVNRDIVETE.....QNSHAGQHKLALSVGESIVIVDTKDIIY    155

HD73_5855    VGLVDGKVTVKTTRETYVTHDTLVILEKKLPQASFMRVHRSFIANINHITEIQPWFNSTYNLIMKEGSKVPVSRTYAKEL    240
SAV0261      IGTHNGITTIHTTNHKYETTEPLNRYEKRLNPTYFIRIHRSYIINTKHIKEVQQWFNYTYMVILTNGVKMQVGRSFMKDF    239
BSU28920     AGTEDGHVNVKTFDHSYTVSDTLVVIEKKLPDSDFIRVHRSFVVNTEYIKEIQPWFNSTYNLIMKDGSKIPVSRTYAKEL    235

HD73_5855    KKLLRI                                                                              246
SAV0261      KASIGL                                                                              245
BSU28920     KKLLHI                                                                              241
```

LrgA/YsbA:

```
HD73_5854    ....MSTKKVYSFLSQAFIFSAIMLISNIIATHLPIPMPSSVIGLVILFSLLCLKVIKLEQVESLGTALTGIIGFLFVPS    76
SAV0262      MKQQKDASKPAHFFHQVIVIALVLFVSKIIESFMPIPMPASVIGLVLLFVLLCTGAVKLGEVEKVGTTLTNNIGLLFVPA    80
BSU28910     ....MSAKKVYGFLTQAFIFAVIMLVSNMIAAIVPIPIPASVVGLVLLFLLLCLKVIKLEQVETLGTSLTSLIGFLFVPS    76

HD73_5854    GISVINSLGVMGQYFVQILTVIVVATVILLAVTGLFAQFILGKDEKETEDTKELKVVNKGRKHGKVA..           143
SAV0262      GISVVNSLGVISQAPFLIIGLIIVSTILLLICTGYVTQIIMKVTSRSKGDKVTKKIKIEEAQAHD....           145
BSU28910     GISVMNSLGVMQQYGLQIVLVILLATIILLGATGLFSQLILSLSGKRKTEADMKTKTVQSPQNNNELVH           145
```

LrgB/YsbB:

```
HD73_5853    ...MASTMTPYFGIVVSLIAYGIGTLLFKHSKGFFLFTPLFVAMVLGIVFLKVGNFTFEEYNTGGKMISFFLEPATIAFA    77
SAV0263      MINHLALNTPYFGILLSVIPFFLATILFEKTNRFFLFAPLFVSMVFGVAFLYLTGIPYKTYKIGGDIIYFFLEPATICFA    80
BSU28900     ...MESTMSPYFGIVVSLAAFGIGTFLFKKTKGFFLFTPLFVAMVLGIAFLKIGGFSYADYNNGGEIIKFFLEPATIAFA    77

HD73_5853    IPLYKQVDKLKKYWWQILSAIVVGSICSVIVVFIVAKAIGLDTAVMNSMLPQAATTAIALPISESIGGIPAITSFAVIFN    157
SAV0263      IPLYKKREVLVKHWHRIIGGIGIGTVVALLIILTFAKLAQFANDVILSMLPQAATTAIALPVSAGIGGIKELTSLAVILN    160
BSU28900     IPLYKQRDKLKKYWWQIMASIIAGSICSVTIVYLLAKGIHLDSAVMKSMLPQAATTAIALPLSKGIGGISDITAFAVIFN    157

HD73_5853    AVIVYALGALFLKTFRVKHPIAKGLALGTAGHALGVAVGIEMGEVEAAMASIAVTVVGVVTVVVIPMFIPFIG        230
SAV0263      GVIIYALGNKFLKLFRITNPIARGLALGTSGHTLGVAPAKELGPVEESMASIALVLVGVVVAVVPVFVAIFF        233
BSU28900     AVIVYALGALFLKVFKVKNPISKGLALGTSGHALGVAVGIEMGEVEAAMASIAVVVVGVVTVLVIPVFVQLIG        230
```

Supplementary Figure 1. Alignments of LytSR and LrgAB from *Bacillus thuringiensis*, *Staphylococcus aureus*, and *Bacillus subtils*. Black background indicated the consensus sequence.

Supplementary Figure 2

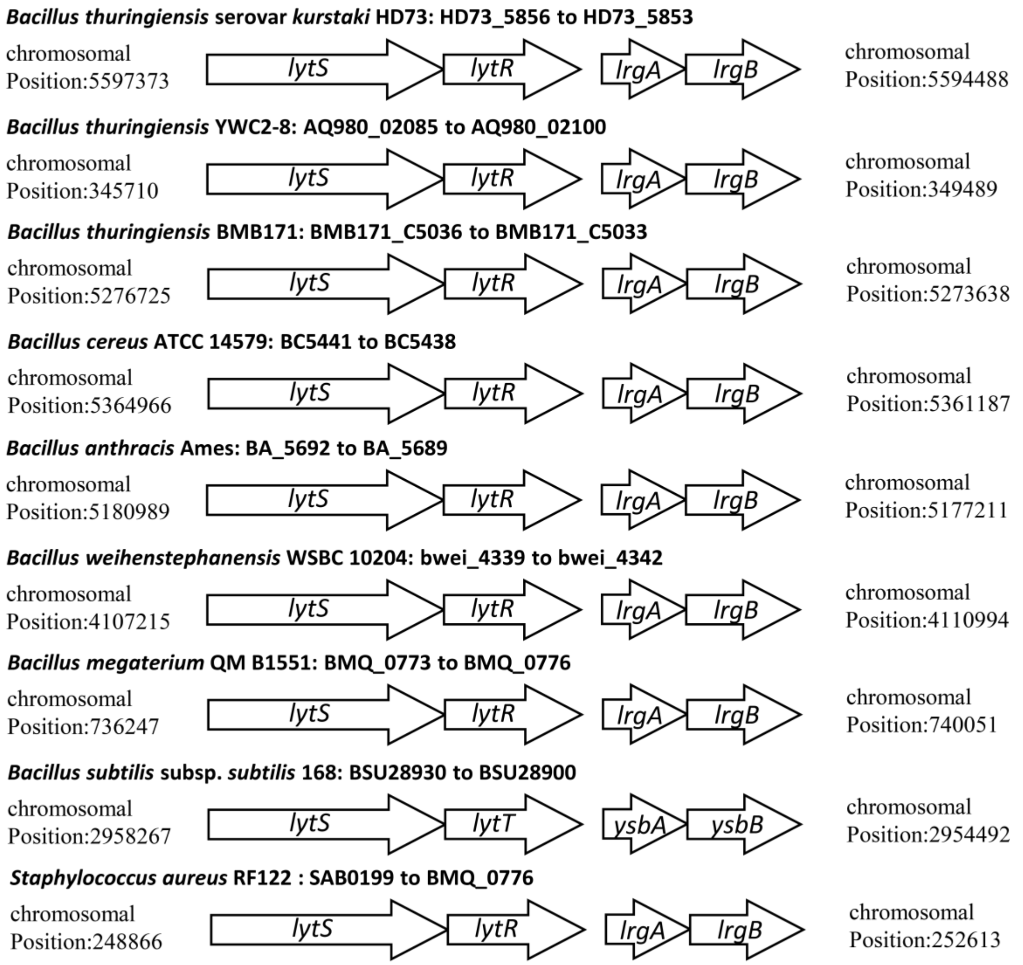

Supplementary Figure 2. The structure of the *lytSR* and *lrgAB* operons in different strains. The corresponding strains and the ORFs of each locus are indicated above their structure.
